# Anterior inferior cerebellar artery aneurysm with proximal parent artery stenosis: A case report

**DOI:** 10.1016/j.radcr.2023.11.090

**Published:** 2023-12-21

**Authors:** Masahiro Morishita, Hidekazu Takada, Takaaki Yamazaki, Hiroshi Moriwaki, Makoto Senoo, Mikio Nishiya

**Affiliations:** Department of Neurosurgery, Hakodate Neurosurgical Hospital, Hakodate, Hokkaido, Japan

**Keywords:** Anterior inferior cerebellar artery, Cerebral aneurysm, Coil embolization, Proximal artery stenosis

## Abstract

Hemodynamic factors are associated with the progression of cerebral aneurysms. We report a 78-year-old woman with an anterior inferior cerebellar artery aneurysm and proximal stenosis of the anterior inferior cerebellar and basilar arteries. The aneurysm exhibited growth on annual follow-up imaging. Aneurysmal rupture occurred 4 years after diagnosis. Coil embolization resulted in aneurysm occlusion with parent artery preservation. Aneurysms adjacent to a more proximal region of severe stenosis in the parent vessel should be considered at high risk for growth or rupture. Such aneurysms require careful monitoring. Particular attention should be paid to posterior circulation aneurysms that occur at anatomically vulnerable sites.

## Introduction

Cerebral aneurysms occur in 3%-5% of the general population [1]. Their rupture can cause subarachnoid hemorrhage, which is associated with high morbidity and mortality. Age, hypertension, history of subarachnoid hemorrhage, aneurysm size, aneurysm location, and geographical location are known risk factors for aneurysmal rupture [2]. Hemodynamic factors have been associated with the progression of cerebral aneurysms. Proximal artery stenosis may have a role in aneurysmal growth and rupture [3], [4], [5]. However, few posterior circulation aneurysms located distal to a region of stenosis have been reported [6]. We report a patient with such an aneurysm on the anterior inferior cerebellar artery (AICA) who was followed for 4 years before rupture.

## Case report

**Fig. 1:**
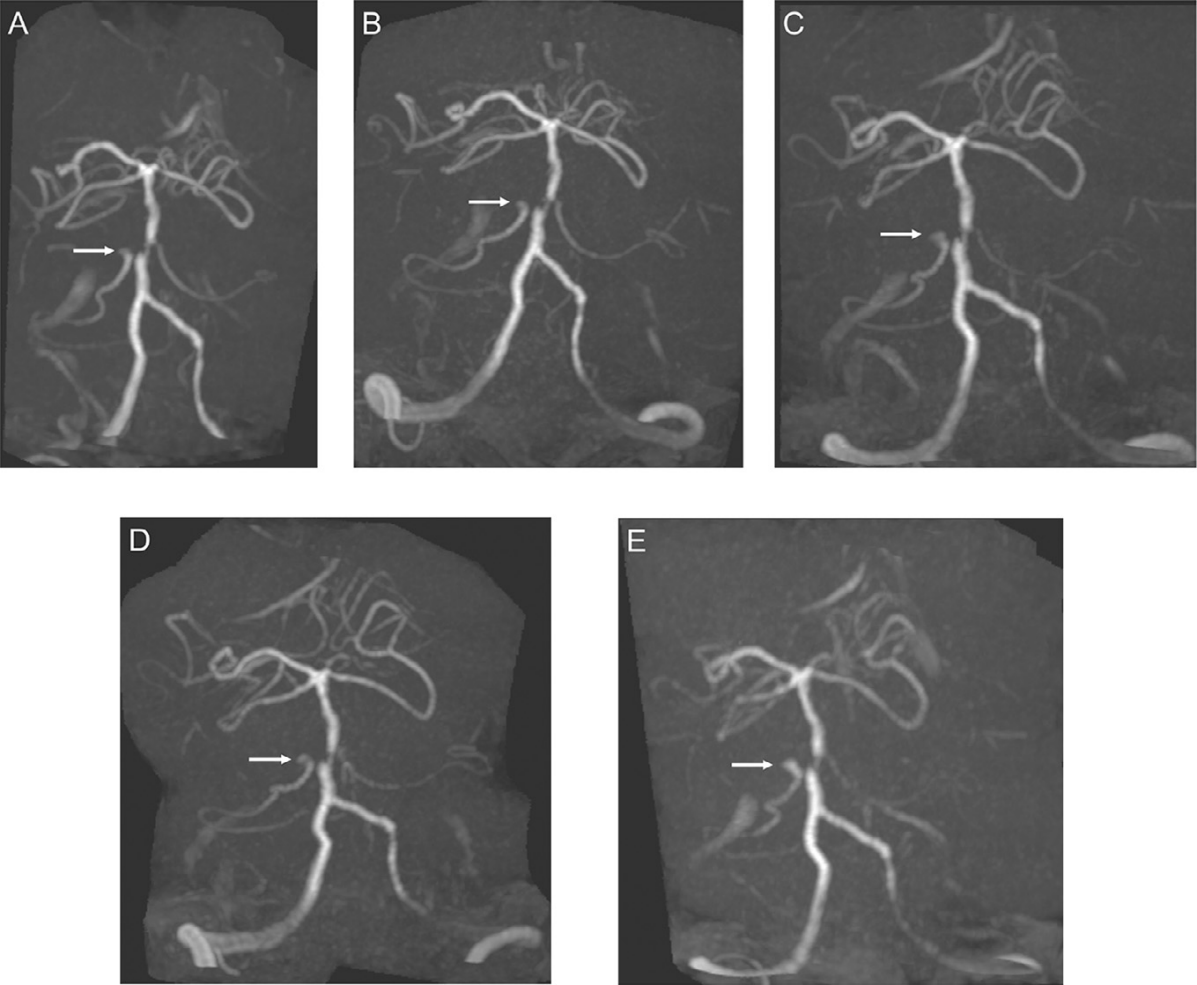
(A) Magnetic resonance angiography showing an anterior inferior cerebellar artery aneurysm (arrow) with proximal anterior inferior cerebellar artery and basilar artery stenosis. (B–E) The aneurysm (arrow) grew from 2 mm to 3 mm over 4 years.

**Fig. 2:**
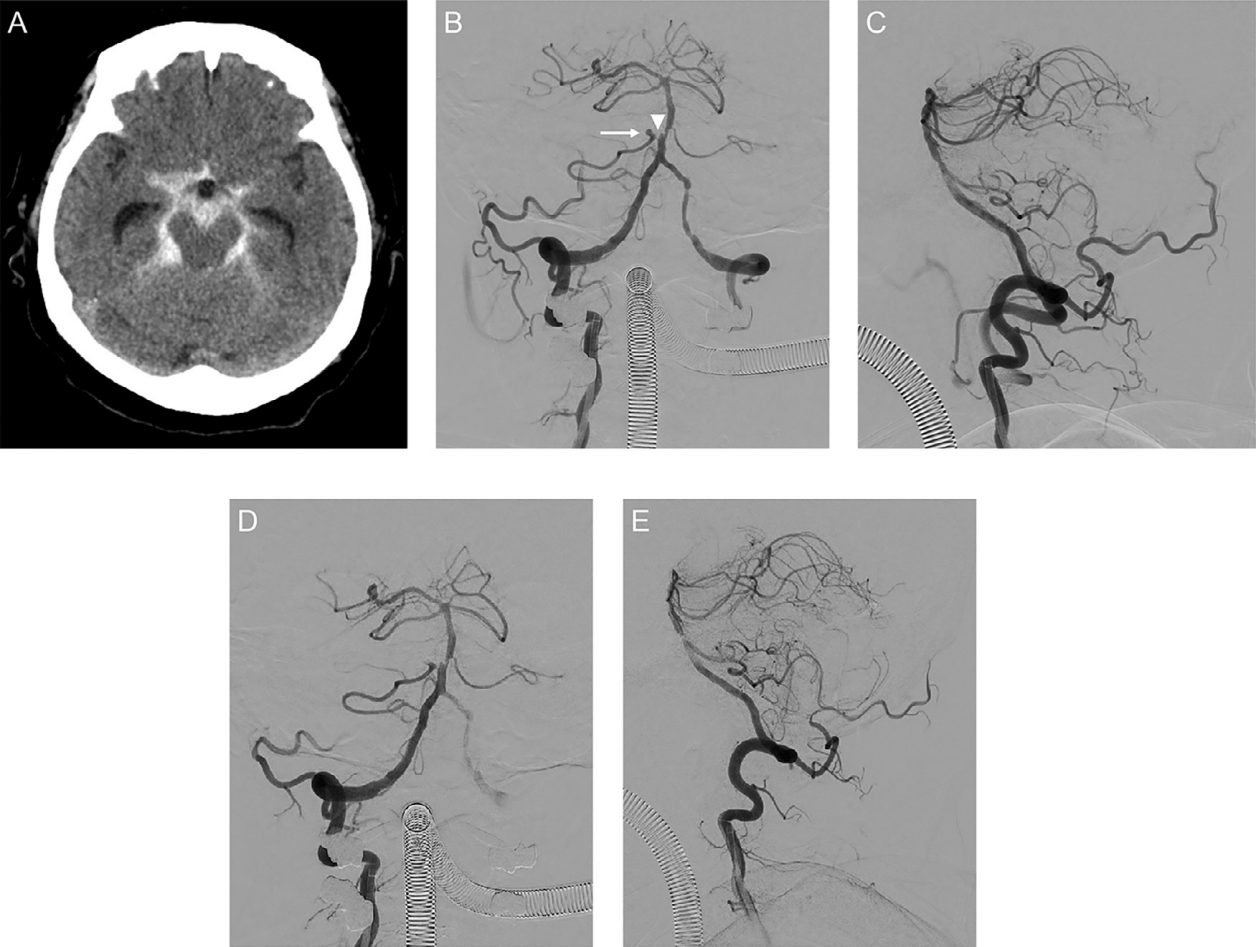
(A) Computed tomography showing subarachnoid hemorrhage. Posteroanterior (B) and lateral (C) views of left vertebral digital subtraction angiography showing a 3 mm aneurysm of the anterior inferior cerebellar artery along an arterial bend (arrow). Stenosis of the proximal parent artery and the basilar artery are also apparent (arrow heads). The ipsilateral posterior inferior cerebellar artery is hypoplastic (dotted arrow). Posteroanterior (D) and lateral (E) views of left vertebral digital subtraction angiography after coil embolization showing occlusion of the aneurysm and preservation of the parent artery.

A 78-year-old woman with a history of hypertension, diabetes mellitus, and hyperlipidemia was referred for evaluation of a right AICA aneurysm diagnosed on magnetic resonance angiography. Stenosis was apparent in the parent artery proximal to the aneurysm as well as the basilar artery (Fig. 1A). She was treated with antihypertensive, antidiabetic, and lipid-lowering drugs. Serial imaging demonstrated annual aneurysmal growth (Figs. 1B–E). Four years after the initial visit, the patient presented to the hospital with a thunderclap headache. Neurological examination was notable only for neck stiffness (Hunt and Hess grade II). Computed tomography showed subarachnoid hemorrhage (Fig. 2A). Digital subtraction angiography showed a 3 mm right AICA aneurysm (Figs. 2B and C). Coil embolization was performed under general anesthesia. After administration of intravenous heparin, a 4 F Fubuki guiding sheath (Asahi Intec, Aichi, Japan) was positioned in the V2 segment of the right vertebral artery and a 3.2 F Tactics microcatheter (Technocrat Corp., Aichi, Japan) was advanced as a distal access catheter. Under road map guidance, an Excelsior SL-10 microcatheter (Stryker, Fremont, CA) was placed into the aneurysm with the assistance of a 0.014-inch Chikai microguidewire (Asahi Intec). The aneurysm was then embolized using multiple i-ED SilkySoft coils (Kaneka, Osaka, Japan). The final angiogram showed adequate occlusion of the aneurysm with preservation of the right AICA (Figs. 2D and E).

## Discussion

We report a rare case of AICA aneurysm associated with proximal artery stenosis. The aneurysm ruptured after 4 years of follow-up. Arterial stenosis can produce high wall shear stress in distal vessels and may be involved in distal aneurysm formation [3]. Proximal artery stenosis has been associated with aneurysm recurrence after clipping and endovascular embolization [4]. Moreover, the severity of stenosis and its distance from the aneurysm neck have been associated with risk of rupture [5]. Aneurysms located near a more proximal region of severe stenosis, as in our patient, should be considered at high risk of growth and rupture.

AICA aneurysms can be categorized according to location as proximal, meatal, and distal. Proximal ones arise from the AICA origin, premeatal segment, or AICA bifurcation [7]. The aneurysm in our patient was located on the premeatal segment. The anatomical features of premeatal segment aneurysms have not been well characterized; however, in our patient and in several previous reports [8], [9], [10], [11], the ipsilateral posterior inferior cerebellar artery was hypoplastic or aplastic, which may have caused increased hemodynamic stress on the AICA. In another report, the premeatal segment aneurysm was located along an arterial bend, a region congenitally vulnerable to aneurysm development [12]. Either feature may underly development of AICA aneurysms at nonbranching sites.

Although the annual rupture rate for small aneurysms (≤5 mm) is quite low (<0.5%), small aneurysms paradoxically account for up to 35% of all ruptured aneurysms [13,14]. Identifying which small aneurysms have a higher rupture risk would help guide treatment decision making. In addition to the known risk factors of age, hypertension, history of subarachnoid hemorrhage, aneurysm size, aneurysm location, and geographical location, rupture risk has also been associated with aneurysm growth. A 2016 meta-analysis of unruptured aneurysms reported that the aneurysmal rupture rate was significantly higher for growing aneurysms than stable ones (3.1% vs 0.1%) [15]. Furthermore, case-specific risk factors such as anatomic vulnerability, as in our patient, probably play a role as well.

Because most recurrences occur within the first 6 months of coil placement, imaging follow-up is initially performed 3-6 months after treatment [16,17]. Then, it is continued for at least 2 years, although the optimal follow-up period is unknown [18]. Magnetic resonance angiography is a suitable noninvasive modality; however, when recanalization is observed, digital subtraction angiography should be performed to confirm and guide further treatment. Careful follow-up is essential even when angiographic complete occlusion has been achieved, as such aneurysms can still recanalize and recur.

## Conclusion

Aneurysms adjacent to a more proximal region of severe stenosis in the parent vessel should be considered at high risk for growth or rupture. Such aneurysms require careful monitoring. Particular attention should be paid to posterior circulation aneurysms that occur at anatomically vulnerable sites.

## IRB approval

This study was approved by the institutional review board.

## Author contributions

Masahiro Morishita is responsible for project development, data collection and analysis, literature research, and manuscript writing. Hidekazu Takada is responsible for literature research and manuscript editing. Takaaki Yamazaki, Hiroshi Moriwaki and Makoto Senoo are responsible for data collection and management. Mikio Nishiya is responsible for project development and total management.

## Ethical statement

All procedures performed in studies involving human participants were in accordance with the ethical standards of the institution and/or national research committee and with the 1964 Helsinki Declaration and its later amendments or comparable ethical standards. The study was approved by the Ethics Committee of Hakodate Neurosurgical Hospital (No. 23-09-01).

## Patient consent

An informed consent was obtained from the patient.
